# Prosthetic Rehabilitation of a Female With Bilateral Transfemoral Amputation in Japan: A Case Report

**DOI:** 10.7759/cureus.46566

**Published:** 2023-10-06

**Authors:** Yohei Tanaka, Takaaki Ueno

**Affiliations:** 1 Rehabilitation Medicine, JR Tokyo General Hospital, Tokyo, JPN

**Keywords:** lower-limb prostheses, stubby prostheses, microprocessor-controlled prosthetic knee, female amputee, prosthetic rehabilitation, bilateral transfemoral prostheses, bilateral transfemoral amputation

## Abstract

Individuals with bilateral transfemoral (TF) amputation experience difficulties when walking with lower limb prostheses. Walking with prostheses is even more difficult when the cause of the amputation is nontraumatic, or the gender is female. We provided prosthetic rehabilitation to a woman who underwent bilateral TF amputation due to internal disease.

A 42-year-old woman underwent bilateral TF amputation for ischemic necrosis of the lower extremities during septic shock treatment. Upon admission to our convalescent rehabilitation ward 3 months after surgery, the patient weighed 32 kg and was underweight. After admission, she underwent strength training of the trunk and hip muscles, hip joint range of motion exercises, and bottom shuffle exercises on the physical therapy table. The prosthetist created stubby prostheses for standing and standing-up exercises on the floor, as well as gait exercises. We gradually extended the length of her prostheses and subsequently switched her knee joints to Ottobock locking and polycentric knees and eventually to Kenevo, which are microprocessor-controlled prosthetic knees (MPK). During occupational sessions, she practiced household activities such as washing dishes, cleaning, and cooking while wearing her prostheses. Six months after admission, the patient was discharged and could walk outdoors alone with two canes without using a wheelchair. At discharge, the Kenevo modes were Mode C on the right and Mode B + on the left. The patient's weight recovered to 41 kg. The patient completed the 10-meter walk test at 0.50 m/s at a comfortable walking speed, the 6-minute walk test at 180 meters, and the timed up and go (TUG) test in 26 seconds. The motor Functional Independence Measure (FIM) score was improved from 60 on admission to 83 on discharge.

Strengthening the hip and trunk muscles, improving endurance and balance, preventing hip contracture, and maintaining the hip range of motion are necessary for walking with bilateral TF prostheses. In the prosthetic rehabilitation of bilateral TF amputations, stubby prostheses, protocols for gradual extension of the prosthetic length, and Kenevo, a mode-changeable MPK, are helpful. MPK is essential for individuals with bilateral TF amputations to walk independently and use their prostheses daily. This report is a valuable reference for healthcare professionals involved with bilateral TF amputees in the future who need prosthetic rehabilitation.

## Introduction

A previous study demonstrated that the gait acquisition rate with lower-limb prostheses was 50.0% for unilateral transtibial amputees and 20.0% for unilateral transfemoral (TF) amputees [1]. TF amputees encountered greater difficulty walking with lower-limb prostheses compared to transtibial amputees. While this previous study specifically examined unilateral amputees, it is evident that bilateral TF amputees face even more significant difficulties in gait acquisition than unilateral TF amputees.

In the case of bilateral TF amputees, even if they can walk with their lower limb prostheses, the walking speed is slow and energy expenditure is significant [2,3]. Therefore, while bilateral TF amputees, with trauma as the cause of amputation can become ambulatory, there are reports of bilateral TF amputees with chronic limb-threatening ischemia (CLTI) who experience difficulty walking and aim for a wheelchair [4]. A 41-year-old woman with bilateral TF amputation due to trauma could walk with lower limb prostheses but preferred a wheelchair because of high energy expenditure when walking [5]. This suggests that even if the amputation is due to trauma, it is difficult for a woman to live without a wheelchair.

With recent advances in technology and rehabilitation, it is now possible for even bilateral transfemoral amputees to achieve practical gait [6]. However, few reports on rehabilitation programs help patients walk with their lower limb prostheses.

We provided prosthetic rehabilitation using a microprocessor-controlled prosthetic knee (MPK) to a female with bilateral TF amputation in her 40s. Finally, the patient was able to walk independently and was discharged. Here, we report on the process of this rehabilitation treatment. Prosthetic rehabilitation was performed in a convalescent rehabilitation ward in the Japanese medical system.

## Case presentation

Here, we present the case of a 42-year-old woman. The patient developed ischemic necrosis of both lower extremities during pneumonia and septic shock treatment, which led to TF amputation. She had a history of anorexia nervosa but no history of diabetes or vascular disease. Three months after surgery, after her general condition improved, she was admitted to our hospital's convalescent rehabilitation ward for prosthetic rehabilitation.

On admission, the patient weighed 32 kg, and the body mass index was 12.5, indicating a significant underweight condition. Before the amputation, the patient's height was 160 cm. Her hip range of motion was 80° in flexion and 15° in extension on both sides. Her lower-extremity muscle strength was level 3 bilaterally on manual muscle testing (MMT). The residual limb lengths were 21 and 22 cm on the right and left sides, respectively.

**Figure 1:**
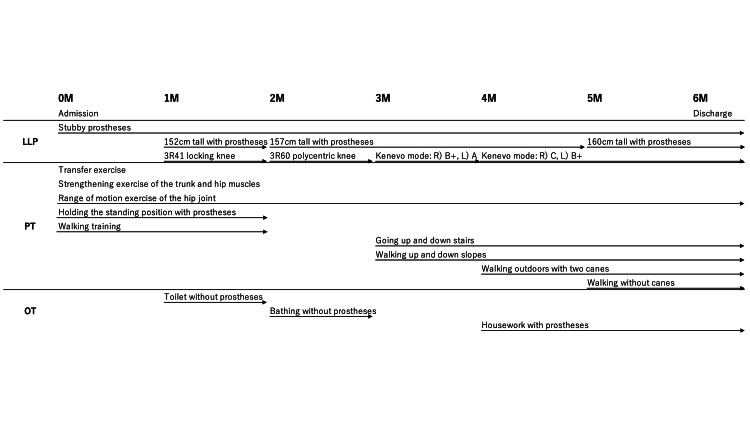
Progress in prosthetic rehabilitation LLP, lower-limb prostheses; PT, physical therapy; OT, occupational therapy; M, months; R, right; L, left

The progress of prosthetic rehabilitation is shown in Figure 1. She received daily rehabilitation for 2-3 hours. Upon admission, she underwent strength training of the trunk and hip muscles, hip joint range of motion exercises, and bottom shuffle exercises on the physical therapy table. We also ensured safe transfers to and from the wheelchair.

The prosthetist created the stubby prostheses to perform standing and standing-up exercises on the floor (Video 1), strength training (Video 2), and gait training (Video 3).

**Video 1:** Standing-up exercises on the floor using stubby prostheses

**Video 2:** Strength training using stubby prostheses

**Video 3:** Gait training using stubby prostheses

We gradually increased the length of her prostheses starting 1 month after admission (Video 4) and incorporated the Ottobock 3R41 locking knee joints into the prostheses (Video 5). The height of the prosthesis was 152 cm. Similar to stubby prostheses, rehabilitation consists mainly of standing and walking with lower limb prostheses. She started training in daily living activities, such as toileting, in occupational therapy.

**Video 4:** Stubby prostheses with slightly extended prosthetic length

**Video 5:** Gait training using 3R41 locking knee joints

Two months after the admission, the length of her prosthesis was extended to 157 cm. At this time, the knee joints were bilateral Ottobock 3R60 polycentric knees with a bouncing mechanism, which locks the knee joint after slight flexion during the initial contact in the stance phase [7], also called stance phase flexion. In the case of the 3R60, the knee joint flexes 15 degrees at initial contact during the stance phase and then locks to prevent further bending. The advantage of this mechanism is that it is easier to descend slopes than if the knee joint were fixed entirely. The patient was fitted with these knee joints on both sides and trained to walk with the lower limb prostheses using two Lofstrand crutches (Video 6). However, she feared that these knee joints might cause her to fall because of knee collapse. During this period, occupational therapy involved shower bathing, which the patients could perform alone.

**Video 6:** Gait training using 3R60 polycentric knee joints

Kenevo is the MPK manufactured by Ottobock for low-to-medium-activity TF amputations. The knee joint has four modes. In mode A, the knee joint is locked and does not initiate the swing phase. In mode B, the knee joint is locked during the stance phase. It unlocks when users begin swinging their legs during the swing phase. Mode B+ corresponds to mode B but is supplemented by a stance phase flexion at a heel strike of up to 10°. This function corresponds to a bouncing mechanism, which allows users to move naturally down slopes. As soon as the heel hits the ground, the knee bends slightly. Mode C corresponds to the yielding mode. The yielding mode is microprocessor-controlled for stance yielding [8]. Stance yielding, usually provided by a hydraulic cylinder, offers resistance to prosthetic knee flexion during stance phase loading, which helps activities that require controlled lowering of the body center of mass, such as sitting on a chair, walking down a ramp, and stair descent [8,9]. The patient's gait training began with the right side set to Mode B+ and the left side set to Mode A (Video 7). She practiced sitting on a chair, ascending and descending stairs, and walking up and down slopes.

**Video 7:** Gait training using Kenevo MPK knee joints (Right: Mode B+, Left: Mode A) MPK: microprocessor-controlled prosthetic knee

Four months after admission, the Kenevo mode was set to Mode C on the right side and Mode B+ on the left. We actively practiced walking up and down the slopes to help her get accustomed to the Mode C yielding function (Video 8). Around this time, she began outdoor walking training to become accustomed to outdoor environments. The patient walked supported by two canes. During occupational therapy, she practiced household activities such as washing dishes, cleaning, and cooking while wearing the prostheses. She also practiced moving barefoot around her home (Japanese style) and carrying objects while walking.

**Video 8:** Walking up and down the slope using Kenevo MPK knee joints (Right: Mode C, Left: Mode B+) MPK: microprocessor-controlled prosthetic knee

Five months after admission, we attempted to change the mode of Kenevo to Mode C on both sides, but the patient was too afraid of falling to continue using it; therefore, we decided to continue with Mode C on the right and Mode B+ on the left. At this time, she could walk without a cane for short distances (Video 9) and perform ascending and descending training on the steeper slopes (Video 10) and stairs with more steps.

**Video 9:** Walking without a cane using Kenevo MPK knee joints (Right: Mode C, Left: Mode B+) MPK: microprocessor-controlled prosthetic knee

**Video 10:** Ascending and descending training on the steeper slopes using Kenevo MPK knee joints (Right: Mode C, Left: Mode B+) MPK: microprocessor-controlled prosthetic knee

Six months after admission, the prosthetist completed the lower-limb prostheses with definitive sockets, and the patient was discharged and could walk outdoors alone with two canes. She was independent in her indoor and outdoor activities of daily living (ADLs) and lifestyle. She could live without a wheelchair.

**Figure 2:**
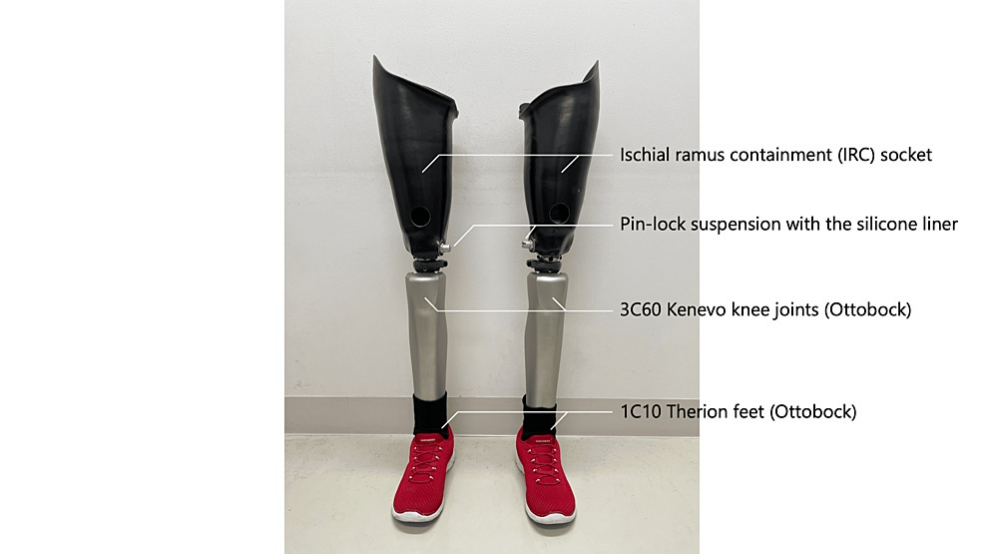
Prescription of the bilateral TF prostheses TF: transfemoral

The prescribed prosthetic configuration was an ischial ramus containment (IRC) socket, a pin-lock suspension with a silicone liner, 3C60 Kenevo knee joints (Ottobock), and 1C10 Therion feet (Ottobock) bilaterally (Figure 2). The final height was set at 160 cm under prosthetic wear.

At discharge, her weight had recovered to 41 kg. Her hip range of motion improved to 120° of flexion on both sides. There was no significant change in hip extension range of motion. She completed the 10-meter walk test [10] at 0.50 m/s at a comfortable walking speed; the 6-minute walk test [11] at 180 meters; and the timed up and go (TUG) test [12] at 26 seconds.

**Figure 3:**
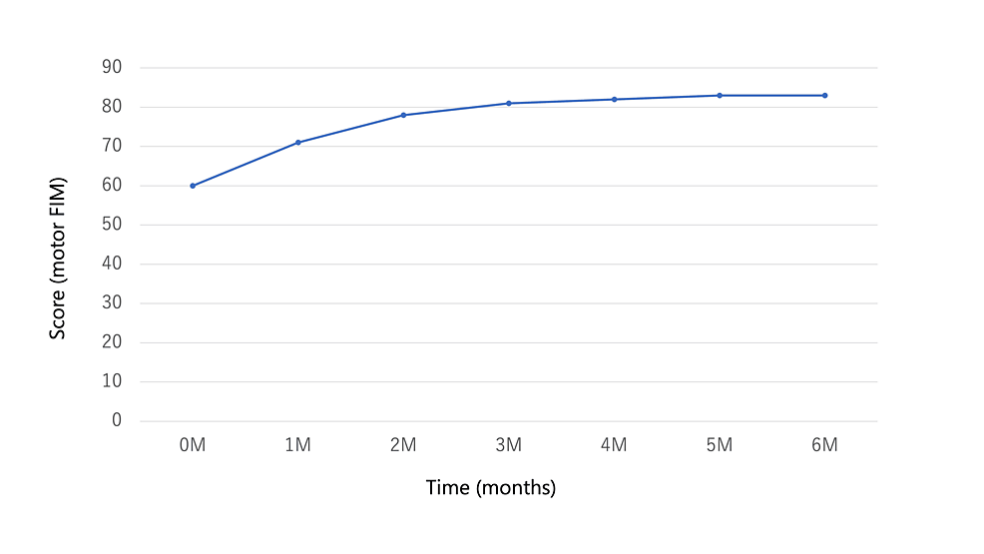
Motor FIM FIM: Functional Independence Measure; M, month

**Figure 4:**
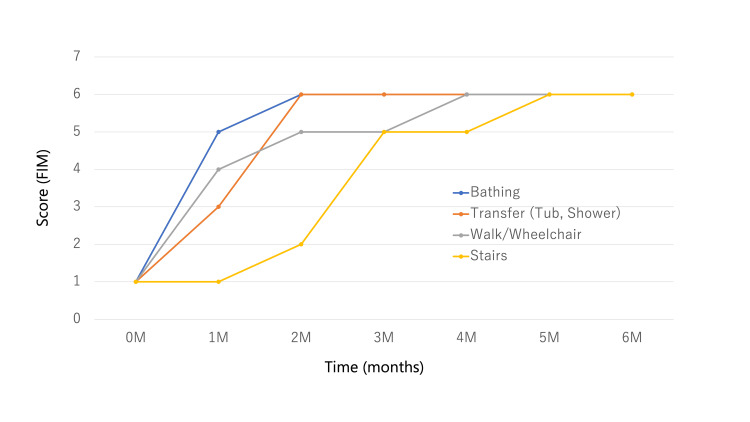
FIM items with significant improvement FIM, Functional Independence Measure; M, month

We assessed the ADLs using the Functional Independence Measure (FIM) [13] during hospitalization. We graphed the progress of the motor FIM (Figure 3) and FIM items with significant improvement (Figure 4). The cognitive FIM had a perfect score of 35 on admission, indicating normal cognitive function. Motor FIM score improved from 60 at admission to 83 at discharge. The full score for the Motor FIM was 91. The Motor FIM, bathing, transfer, walk, and stairs were significantly improved over time.

After 2 months of continuing gait training, she could switch from Kenevo mode to Mode C bilaterally. She also took stubby prostheses home for muscle strength and gait training (Video 11). Two years after the surgery, she could walk outdoors and ride a train independently.

**Video 11:** Gait training using stubby prostheses at discharge

## Discussion

Bilateral TF amputees require the following to acquire the ability to walk with lower limb prostheses: hip strength [14], hip range of motion, trunk strength, endurance, and balance [15]. In addition to these basic physical abilities, they must be able to rise from the floor, maintain a standing position, walk, and perform complex tasks such as climbing hills and stairs.

The patient underwent a stepwise extension of the length of the lower limb prostheses, which is a practical approach for bilateral TF amputees [16]. However, there are no clear guidelines for setting the prosthesis length for bilateral TF amputees [15]. Thus, as we gradually increased the length of the patient's prostheses, we set it to the length at which the patient could stand and walk with her prostheses without fear and with confidence.

Technological advancements have contributed to the success of bilateral TF prostheses. Compared to transtibial prostheses, TF prostheses are more challenging to use because of the loss of the knee joint, which leads to difficulties in descending stairs or slopes and a higher risk of falling. Conventional mechanical knee joints cannot address this problem, whereas MPK can. The microprocessor controls the resistance of knee bending in the stance and swing phases based on sensor information built into the lower-limb prostheses [17]. MPKs have been shown to reduce the risk of uncontrolled falls by up to 80% [18]. Adjusting the flexion resistance of the knee joint in the stance phase to match the user's gait cycle and movements is essential for preventing falls. This is similar to the eccentric contraction of the quadriceps muscles in healthy people. When the quadriceps engage in eccentric contractions, they function as shock absorbers [19]. This feature of the MPK prevents the knee from bending too abruptly, not only when walking but also when descending hills and stairs, thus reducing the risk of falling. This function is crucial for walking with prostheses in bilateral TF amputees. 

One of the factors that enabled this patient to walk was the use of MPK knee joints, which allowed bilateral TF amputees to walk with less oxygen consumption [20]. MPKs also reduce energy consumption and improve performance on stairs and slopes compared with conventional mechanical knee joints [21].

Kenevo, an MPK, was selected as the prosthetic knee joint for this case. Depending on the situation, Kenevo can switch between Modes A, B, B+, and C. When using Kenevo for bilateral TF amputees, the final goal is to achieve Mode C with stance yielding on both sides; however, there are two ways to achieve this: Mode C on one side at a time or on both sides simultaneously. To date, no reports have compared these two methods in the prosthetic rehabilitation of bilateral TF amputees. In our experience, simultaneously using the knee joint in mode C on both sides is very difficult. Therefore, it is helpful to temporarily spend some time with Mode C on one side and Mode B+ on the other, as in the present case, and then aim for Mode C bilaterally when physical function improves over an extended period. Kenevo, which can switch modes according to the progress of prosthetic rehabilitation, is advantageous for helping bilateral TF amputees walk.

With the recent introduction of powered knee and ankle prostheses, users of bilateral TF prostheses can now benefit from prosthetic limb parts that assist them with movements such as rising from a chair or climbing stairs [22]. The possibilities for users of bilateral TF prostheses are expanding.

Nevertheless, stubby prostheses, protocols for gradual height increase, and physical therapy to improve basic strength, muscle strength, and balance skills remain essential for bilateral TF amputees to walk with lower limb prostheses.

## Conclusions

In this report, we have described the case of a woman with bilateral TF amputation who could walk with lower limb prostheses as a result of prosthetic rehabilitation. Strengthening the hip and trunk muscles, improving endurance and balance, preventing hip contracture, and maintaining the hip range of motion are necessary for walking with bilateral TF prostheses. In the prosthetic rehabilitation of bilateral TF amputees, stubby prostheses, protocols for gradual extension of the prosthetic length, and Kenevo, a mode changeable MPK, are helpful. MPK is essential for bilateral TF amputees to walk independently and use their prostheses daily. While our findings are promising, further research is needed to explore the impact of different prosthetic components and optimal rehabilitation strategies for this specific population. 
